# Comprehensive analysis of the novel omicron receptor AXL in cancers

**DOI:** 10.1016/j.csbj.2022.06.051

**Published:** 2022-06-27

**Authors:** Wei-Na Zhang, Xue-Ping Li, Peng-Fei Wang, Lu Zhu, Xin-Hua Xiao, Yu-Jun Dai

**Affiliations:** a Department of Hematology, Guangzhou Women and Children's Medical Center, Guangzhou, 510623, China; b Department of Hematologic Oncology, Sun Yat-sen University Cancer Center, Guangzhou 500020, China; c State Key Laboratory of Oncology in South China, Collaborative Innovation Center for Cancer Medicine, Sun Yat-sen University Cancer Center, Guangzhou 510060, China

**Keywords:** COVID-19, coronavirus disease 2019, SARS-CoV-2, severe acute respiratory syndrome coronavirus 2, ACE2, angiotensin-converting enzyme 2, AXL, anexelekto, UMAP, uniform manifold approximation and projection for dimension reduction, TMB, tumor mutation burden, MSI, microsatellite instability, OS, overall survival, DFI, disease-free interval, PFI, progression-free interval, disease-specific survival, TIMER, tumor immune estimation resource, CHOL, cholangiocarcinoma, GBM, glioblastoma multiforme, BLCA, bladder urothelial carcinoma, PAAD, pancreatic adenocarcinoma, LAML, acute myeloid leukemia, LGG, brain lower grade glioma, SKCM, skin cutaneous melanoma, TGCT, testicular germ cell tumors, KICH, kidney chromophobe, BRCA, breast invasive carcinoma, PRAD, prostate adenocarcinoma, THCA, thyroid carcinoma, LIHC, liver hepatocellular carcinoma, STAD, stomach adenocarcinoma, KIRC, kidney renal clear cell carcinoma, KIRP, kidney renal papillary cell carcinoma, LUSC, lung squamous cell carcinoma, COAD, colon adenocarcinoma, UCEC, uterine corpus endometrial carcinoma, HNSC, head and neck squamous cell carcinoma, LUAD, lung adenocarcinoma, ESCA, esophageal carcinoma, MESO, mesothelioma, OV, ovarian serous cystadenocarcinoma, DLBC, lymphoid neoplasm diffuse large b-cell lymphoma, ACC, adrenocortical carcinoma, UVM, uveal melanoma, SARC, sarcoma, PCPG, pheochromocytoma and paraganglioma, Omicron, Cancer, AXL, Immune infiltration, Vaccines, Neoantigens

## Abstract

The SARS-CoV-2 is constantly mutating, and the new coronavirus such as Omicron has spread to many countries around the world. Anexelekto (AXL) is a transmembrane protein with biological functions such as promoting cell growth, migration, aggregation, metastasis and adhesion, and plays an important role in cancers and coronavirus disease 2019 (COVID-19). Unlike angiotensin-converting enzyme 2 (ACE2), AXL was highly expressed in respiratory system cells. In this study, we verified the AXL expression in cancer and normal tissues and found AXL expression was strongly correlated with cancer prognosis, tumor mutation burden (TMB), the microsatellite instability (MSI) in most tumor types. Immune infiltration analysis also demonstrated that there was an inextricable link between AXL expression and immune scores in cancer patients, especially in BLCA, BRCA and CESC. The NK-cells, plasmacytoid dendritic cells, myeloid dendritic cells, as one of the important components of the tumor microenvironment, were highly expressed AXL. In addition, AXL-related tumor neoantigens were identified and might provide the novel potential targets for tumor vaccines or SARS-Cov-2 vaccines research in cancer patients.

## Graphical abstract

**Figure:**
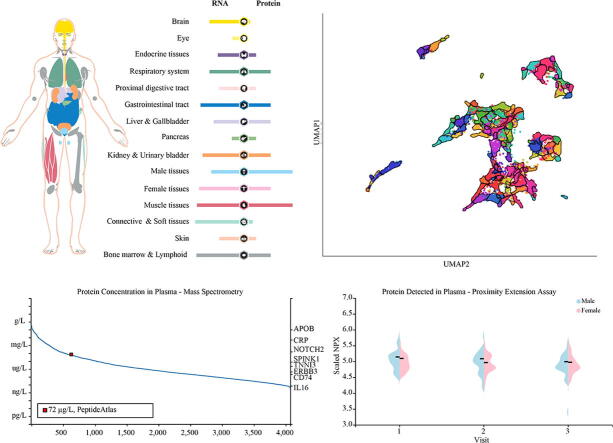

## 1 Introduction

The severe acute respiratory syndrome coronavirus 2 (SARS-CoV-2) broke out in the world in 2019 and had a huge impact on a global scale [1]. Under this circumstance, various vaccines had emerged, hoping to change the current situation [2]. As we know, vaccination against the SARS-CoV-2 related pneumonia was one of the means to reduce the incidence and severe rate of COVID-19, but after vaccination, the antibody level of the human body declined over time, and the protective effect of preventing virus infection also weakened [3].

COVID-19 is multi-organ tropism and could cause fever, cough, severe respiratory illness and multi-organ failures [4]. Host cell receptors were the key determinants of viral tropism and disease initiation [5]. The current mainstream view was that ACE2 was the most important receptor for SARS-CoV-2 to invade the human cells and mainly expressed in the human kidney and digestive system [6]. However, it was only expressed in about 1 in 1,000 lung cells and 2 in 1,000 tracheal cells. Such a low expression of ACE2 receptor protein was difficult to support the high infectivity of SARS-CoV-2 in the population [5]. Therefore, it was speculated that the SARS-CoV-2 might have other important receptors in the human respiratory system.

Recently, anexelekto (AXL) was identified as a novel receptor of omicron [7]. Unlike ACE2, AXL was highly expressed in respiratory system cells, such as type I or II epithelial cells of lung and fibroblasts. The AXL protein on lung cells was combined with spike protein of SARS-CoV-2 and strongly colocalized on the cell membrane [8]. Some studies indicated that there was no cross-infection inhibitory function between ACE2 and AXL, and AXL could independently mediate the omicron infection, which suggested that the AXL protein may be a new omicron receptor that did not depend on the ACE2 protein [7], [9].

Functionally, AXL performed as a critical promoter of cancer cells on immune escape and drug resistance, finally leading to aggressive cancers [10], [11]. Cancer patients always had abnormal immune microenvironment and immune regulation and might be more susceptible to coronavirus infection [4], [12]. Here, we comprehensive analyzed the expression, prognosis and immune infiltration of AXL in cancer patients. This may further improve cancer patients' awareness of SARS-CoV-2 prevention and provide potential clues for the application of vaccines or therapeutic drugs developed by AXL as targets in cancer patients.

## 2 Methods

### 2.1 The Tissue Atlas

The Tissue Atlas database was applied to analyze the transcription and translation expression level of AXL in normal tissues. The expression of AXL in different dataset was visualized in the form of bar graphs. The new version 21 of the Tissue Atlas database could identify the co-expression patterns by using dimensionality reduction strategy and density-based clustering method to explore the landscape of AXL in normal tissues. The relationship of AXL and the co-expression patterns were showed by UMAP clustering. In addition, more than 4072 proteins expression level were also included in this peptide atlas (https://www.proteinatlas.org/humanproteome/blood). The AXL protein concentration in plasma was detected and quantified by mass spectrometry-based proteomics.

### 2.2 Data collection and analysis

The gene expression data of AXL in pan-cancers were obtained from The-Cancer-Genome-Atlas (TCGA) database and GETx database. We used RMA package in R software to filter the comprehensive data, remove the missing or duplicated data and transform the data by log2(TPM + 1). The clinical information of age, clinical stages, sex and tumor stages and tumor mutation burden (TMB) were also obtained from TCGA database. What’s more, it also provided the microsatellite instability (MSI) information of different type of cancers for further analysis. The mutation incidences and insertion or deletion events of AXL were calculated through per million base pair of TMB and MSI. Paired t tests and the *t* test were used to compare AXL expression between tumor and normal tissues.

### 2.3 Cox-regression and survival analysis

The survival data of different type of cancer patients was extracted through TCGA database. Patient samples were divided into low and high groups based on the best separation value of AXL expression. The Kaplan–Meier package was applied to analyze the survival time of patients with different cancer types in the two groups. The observation indicator includes overall survival (OS), disease-free-interval (DFI), progression-free-interval (PFI), disease-specific-survival (DSS) and the correlation between these indicators and AXL expression were examined by Cox regression analysis. In addition, the survival specificity and time-dependent survival sensitivity were detailly analyzed by R package (survival ROC and survival package, rdocumentation.org/packages/survival). The difference between groups and curves were examined by using log-rank test.

### 2.4 Immune infiltration analysis

The immune cell infiltration was analyzed by Tumor Immune Estimation Resource (TIMER, https://cistrome.shinyapps.io/timer/) [13]. The database provided auto-analysis of immune infiltration scores, including neutrophils, dendritic cells, B cells, macrophages, CD4+ and CD8+ T cells and the correlation coefficients were calculated by Pearson. P < 0.05 were considered as significant. All statistics were generated by using ggplot2 and forest-plot packages in R software.

### 2.5 Database of COVID-19

The expression of TAM family genes in healthy individuals and patients with COVID-19 was analyzed by using the data from GEO database (GSE147507, GSE154768 and GSE157103).

## 3 Result

### 3.1 AXL expression in human tissues

**Fig. 1:**
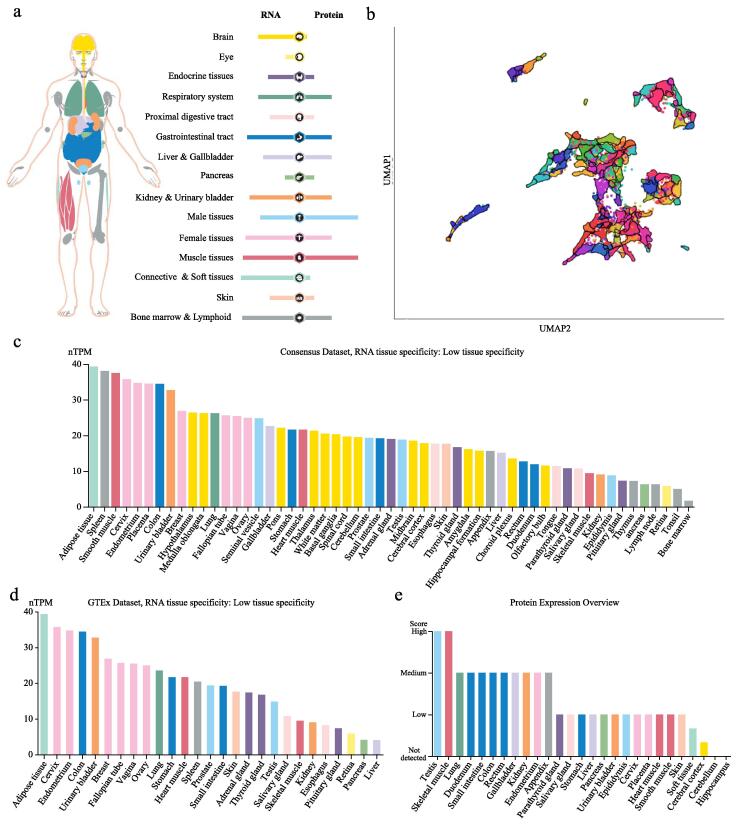
AXL expression in human tissues. (a) The expression and distribution of AXL in human tissues. (b) UMAP plot analysis of AXL expression in tissue clusters and correlation with these clusters. The function and reliability of tissues were also displayed in Table 1. (c) AXL mRNA expression level of human tissues in Consensus dataset. (d) AXL mRNA expression level of human tissues in GTEx dataset. (e) Overview of AXL protein expression in related database.

Here, we explored the AXL RNA and protein expressions in tissues of healthy individuals. We found that AXL at transcriptional level expression was universal expressed in all tissues, including brain, respiratory system, proximal digestive tract, liver/gallbladder, kidney/urinary bladder, male and female tissues and bone marrow/ lymphoid tissues. Whereas, AXL protein expression was mainly expressed in respiratory system, kidney/urinary bladder, bone marrow/lymphoid tissues, gastrointestinal tract, female tissues and more expressed in male tissues, muscle tissues (Fig. 1a). We found 87 gene clusters in all tissue types according to Louvain clustering of AXL expression and calculated and displayed by UMAP (Fig. 1b). AXL was a part of cluster 59 fibroblasts and we identified 15 nearest gene neighbors based on tissue AXL-RNA expression (Table1). Then, the AXL-RNA expression was verified in other datasets. The top 5 tissues with the highest AXL expression in Consensus dataset were adipose tissue, spleen, smooth muscle, cervix and endometrium (Fig. 1c). In the GETx dataset, the order of AXL expression level was as follows: adipose tissue, cervix, endometrium, colon and urinary bladder. Lung was ranked at 12th and 9th in these two datasets respectively (Fig. 1d). Further, the AXL protein expression level showed significant differ from that of RNA expression level and it was highly expressed in testis and skeletal muscle, and more expressed in lung, duodenum, small intestine, colon, rectum, gallbladder, kidney, endometrium and appendix (Fig. 1e).

**Table 1:** Nearest neighbour genes based on tissue AXL-RNA expression in tissues.

Nearest neighbour genes based on tissue AXL-RNA expression in tissues			
Neighbour	Description	Correlation	Cluster
SEMA4C	Semaphorin 4C	0.9333	59
SH3PXD2B	SH3 and PX domains 2B	0.8965	59
ZFP36L1	ZFP36 ring finger protein like 1	0.8386	57
SH3PXD2A	SH3 and PX domains 2A	0.8158	1
LRP1	LDL receptor related protein 1	0.8158	59
ARSI	Arylsulfatase family member I	0.807	1
TGFB3	Transforming growth factor beta 3	0.8	59
PAMR1	Peptidase domain containing associated with muscle regeneration 1	0.786	59
ADCY3	Adenylate cyclase 3	0.7737	59
MMP14	Matrix metallopeptidase 14	0.7667	47
PHLDB1	Pleckstrin homology like domain family B member 1	0.7561	59
CRTC3	CREB regulated transcription coactivator 3	0.7509	59
SKI	SKI proto-oncogene	0.7509	59
COLGALT1	Collagen beta(1-O)galactosyltransferase 1	0.7509	72
NRP2	Neuropilin 2	0.7456	47

### 3.2 Concentration of AXL in plasma

**Fig. 2:**
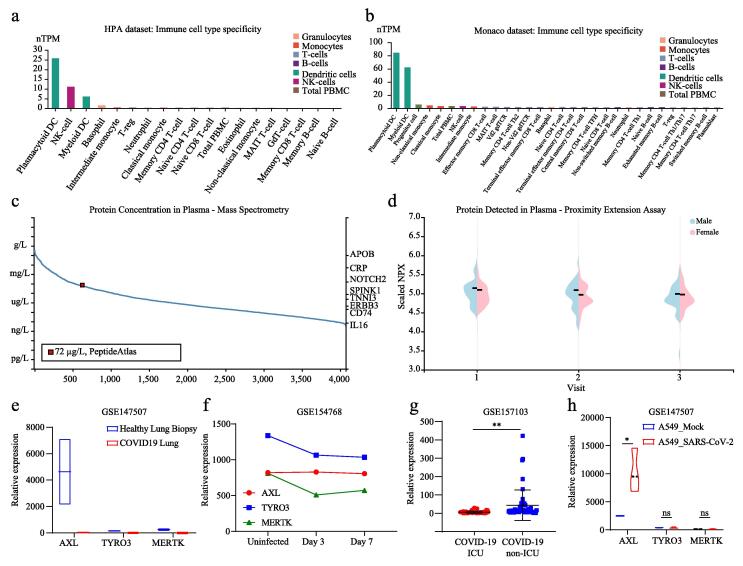
AXL expression profiling in blood. (a) Expression of AXL in human blood immune cells from HPA dataset. (b) Expression of AXL in human blood immune cells from Monaco dataset. (c) The concentration of AXL protein estimated in plasma of peripheral blood. (d) Comparison of AXL protein concentration differs from male and female. (e) TAM family expression level in lung biopsy of healthy individuals and patients with SARS-CoV-2 infection. The data was obtained from GSE147507. (f) The expression of in TAM family genes in samples of individuals with non-infection, 3 days or 7 days after infection by using data from accession number GSE154768. (g) Comparison of AXL expression level between COVID-19 patients in ICU and in non-ICU. (h) The AXL expression alteration in A549 cells infected with mock virus or SARS-CoV-2 through GSE147507. P value, * < 0.05; ** < 0.01; *** < 0.001.

The SARS-CoV-2 not only invaded various organs and tissues of individuals, but also aggravated the infection of patients by affecting the concentration of related proteins in plasma to induce cytokine storms. Cytokine concentrations in plasma were closely related to severity of COVID-19 [14]. Thus, we further explored the AXL protein concentration in plasma. In immune cells, AXL exhibited high expression status in NK-cells (HPA dataset), plasmacytoid dendritic cell, myeloid dendritic cell (HPA and Monaco dataset) (Fig. 2a) and the 15 nearest neighbour genes of AXL were also showed in Table 2. Next, we wondered whether AXL protein in plasma could affect function of these immune cells. The concentration of AXL protein was obtained from the peptide atlas by using mass spectrometry-based proteomics. The AXL protein concentration in plasma was nearly 72 ng/L (Fig. 2b). We also applied proximity extension assay and compared the protein concentration of AXL in plasma based on their gender and found that it was lower in female than that in male in three visits every three month (Fig. 2c).

**Table 2:** Nearest neighbour genes based on tissue AXL-RNA expression in tissues.

Nearest neighbour genes based on tissue AXL-RNA expression in tissues			
Neighbour	Description	Correlation	Cluster
PLS3	Plastin 3	0.8487	3
CTSV	Cathepsin V	0.8323	3
BEND6	BEN domain containing 6	0.8265	47
ADAT3	Adenosine deaminase tRNA specific 3	0.7584	47
CETP	Cholesteryl ester transfer protein	0.7548	47
KRT8	Keratin 8	0.7508	15
UCHL3	Ubiquitin C-terminal hydrolase L3	0.7157	47
TTC24	Tetratricopeptide repeat domain 24	0.7152	3
N4BP2	NEDD4 binding protein 2	0.7019	15
VKORC1L1	Vitamin K epoxide reductase complex subunit 1 like 1	0.701	47
P2RY6	Pyrimidinergic receptor P2Y6	0.6948	47
NBPF20	NBPF member 20	0.6943	3
NLRP7	NLR family pyrin domain containing 7	0.6788	47
PLD4	Phospholipase D family member 4	0.6774	47
MRPL16	Mitochondrial ribosomal protein L16	0.6739	47

### 3.3 Correlation of the TAM family and COVID-19

AXL was within the TAM family (TAM RTKs: *TYRO3*, *AXL*, and *MERTK*) [15]. Thus, we further explored the correlation of the TAM family and COVID-19. Our data indicated that the expression of all these three genes was significantly much lower in lung biopsy samples with COVID-19 compared with these in healthy lung biopsy samples (Fig. 2e). Data from another database (GSE154768) showed the same conclusion. We found that the expressions of these genes were significantly down-regulated after these individuals infected with the virus, but there was no obvious change in these gene expressions on the 7th and 3rd days after infection (Fig. 2f). Further, AXL expression levels were much lower (p = 0.0023) in samples from severely infected patients treated in ICU compared to those non-severely infected samples (Fig. 2g). Then, we investigated these three gene expressions in lung cancer cells (A549) infected with mock virus or SARS-CoV-2. Interestingly, only AXL expression was differentially expressed in the two groups and the AXL displayed a higher expression in A549 with SARS-CoV-2 infection (Fig. 2h). The diametrically opposite results of expression in normal and tumor tissues suggested that other potential mechanisms may exist for AXL in tumors.

### 3.4 Expression and prognosis of AXL in cancers

**Fig. 3:**
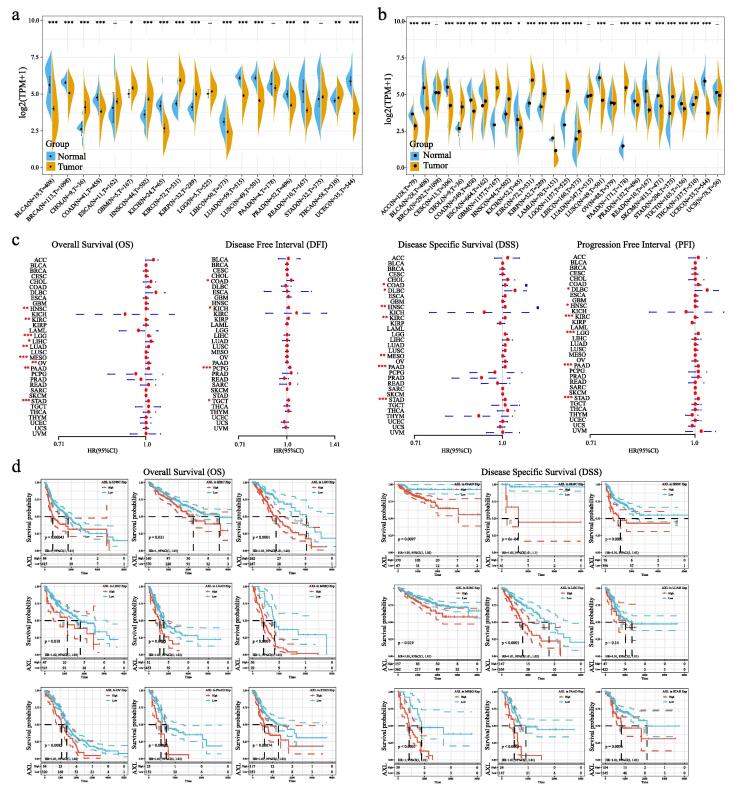
Expression and prognosis of AXL in cancers. (a) Expression of AXL in different types of cancers and adjacent tissues by using TCGA database. (a) AXL RNA expression in more types of cancers and paired normal samples by using TCGA and GETx database. (c) The univariate survival analysis results of AXL expression on OS, DFI, PFI and DSS in all types of cancers. (d) The Kaplan–Meier analysis of AXL expression of AXL expression on OS and DSS in cancers. P value, * < 0.05; ** < 0.01; *** < 0.001.

Tumor patients had limited benefit from SARS-CoV-2 vaccine due to auto-immune disorders [12]. We compared the AXL expression between different type of cancers and corresponding adjacent tissues. In TCGA database, we found AXL was much higher expressed in CHOL, GBM, HNSC, KIRC, KIRP and THCA and lower expressed in BLCA, BRCA, COAD, KICH, LIHC, LUAD, LUSC, PRAD, READ and UCEC (Fig. 3a). Further, we integrated the data of normal tissues in the GETx database to analyze the AXL expression in 27 types of tumors. The results after expanding the sample added a lot of tumor types compared with the previous one, including highly expressed in ESCA, PAAD, STAD and THCA and lowly expressed in LAML, LGG, SKCM and TGCT. In particular, the expression of AXL was significantly elevated in LIHC tumor tissues and was not different in LUAD, which was inconsistent with the results of the TCGA database (Fig. 3b).

Next, we examined the prognosis of AXL in all types of cancers. The univariate survival analysis results of OS indicated that the expression of AXL had obvious significance with prognosis in HNSC, KIRC, LGG, LIHC, MESO, OV, PAAD and STAD. In addition, we found the AXL expression could affect the DFI in COAD, KICH, PCPG and TGCT, and affect the PFI in DLBC, HNSC, KIRC, LGG, PAAD and STAD. The non-tumor-related death factors could affect the survival rate, we further analyzed the relationship between AXL expression and prognosis on DSS. The result showed that the DSS could also be influenced by AXL expression in HNSC, COAD, DLBC, KIRC, MESO, PAAD and STAD (Fig. 3c). The Kaplan–Meier also further validated the above results on OS, DFI, PFI and DSS as showed in Fig. 3d and Supplementary Fig. S1.

### 3.5 Functional analysis of AXL in cancers

**Fig. 4:**
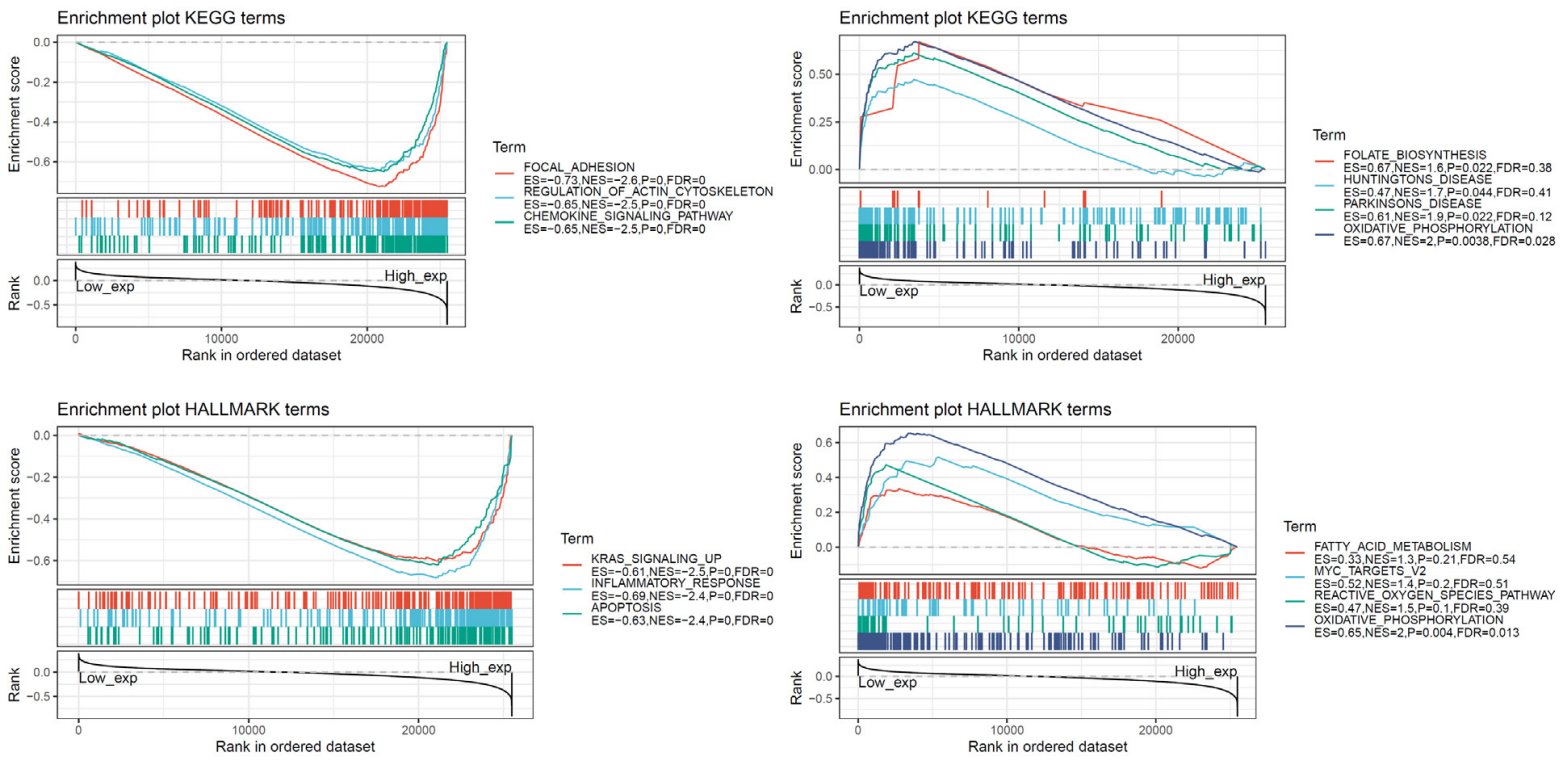
Functional KEGG and Hallmark analysis of differential expressed genes associated with AXL expression.

In order to observe the function of AXL expression in tumors, we divided the samples into high-AXL expression group and low-AXL expression group and applied GSEA to analyze the enrichment of KEGG and HALLMARK between two groups. The most significant top three pathways in higher AXL expression group were focal adhesion, regulation of actin cytoskeleton and chemokine signaling pathway enriched in KEGG terms and KRAS-signaling, inflammatory response and apoptosis pathways enriched in Hallmark terms (Fig. 4). While, the most significant top four pathways in lower AXL expression group were folate biosynthesis, huntingtons disease, parkinsons disease and oxidative phosphorylation pathways enriched in KEGG terms and fatty acid metabolism, MYC target, reactive oxygen species pathway and oxidative phosphorylation pathway enriched in Hallmark terms (Supplementary Table S1).

### 3.6 Immune infiltration of AXL in cancers

**Fig. 5:**
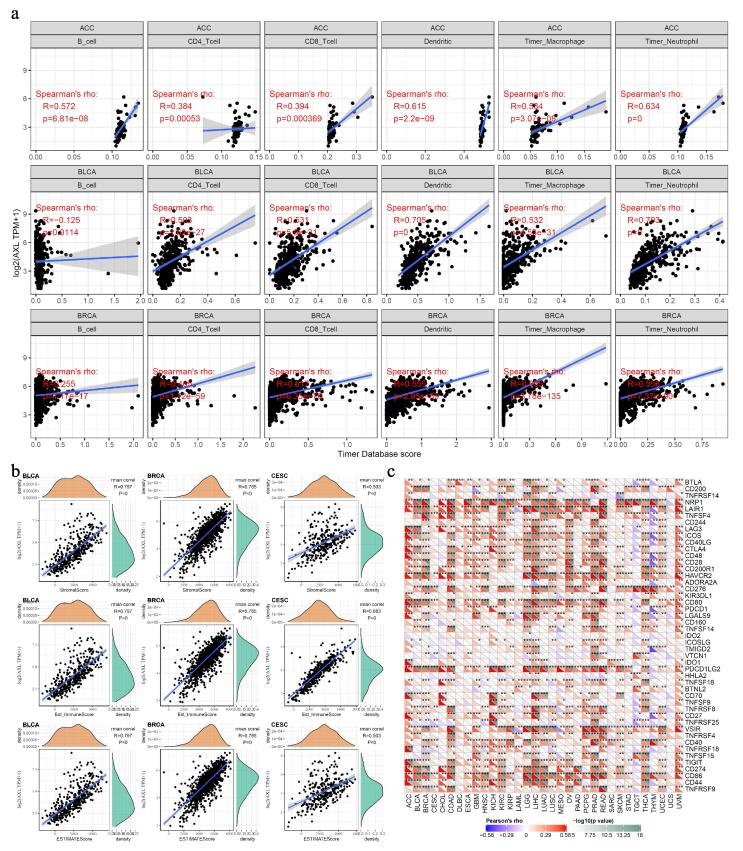
Immune Infiltration of AXL in Cancers. (a) The top 3 most significantly associated tumors correlation with AXL expression and immune cells (B cell, T cell, dendritic cell, macrophage cell and neutrophil cell) by Spearman analysis. (b) The top 3 correlation of AXL expression with immune cell scores in BLCA, BRCA and CESC. (c) The heatmap of correlation between AXL expression and these immune checkpoint genes, P value, * < 0.05; ** < 0.01; *** < 0.001.

Functional analysis indicated chemokine signaling pathway and inflammatory response and apoptosis pathways were associated with AXL expression in cancers. As we known, immune homeostasis and microenvironment in tumor patients were severely disrupted by tumor cells [16]. Tumor-infiltrated lymphocytes were an independent survival predictor in cancers and we investigated if the AXL expression was correlated with immune infiltrated cells in cancers [17]. We utilized the TIMER database to analyze the correlation of AXL expression with immune cell scores in different tumor types [13]. The top 3 most significantly associated tumors were ACC, BLCA and BRCA (Fig. 5a). More and more reports have shown that the tumor immune microenvironment played an important role in the occurrence and development of tumors. Here, we used estimate package to analyze the immune score or stromal score of each sample, and explored the relationship of AXL expression with immune score, stromal score and estimate immune score. Among these 33 types of cancers, the top three most significantly correlated tumors were BLCA, BRCA and CESC (Fig. 5b, Supplementary Fig. S2 and S3). Further, we analyzed the correlation of AXL expression with more than 40 common immune checkpoint related genes. As shown in Fig. 5c, there was a close correlation between AXL expression and these immune checkpoint genes, and most of them showed a positive correlation.

### 3.7 Correlation of AXL with Neoantigen, TMB and MSI

**Fig. 6:**
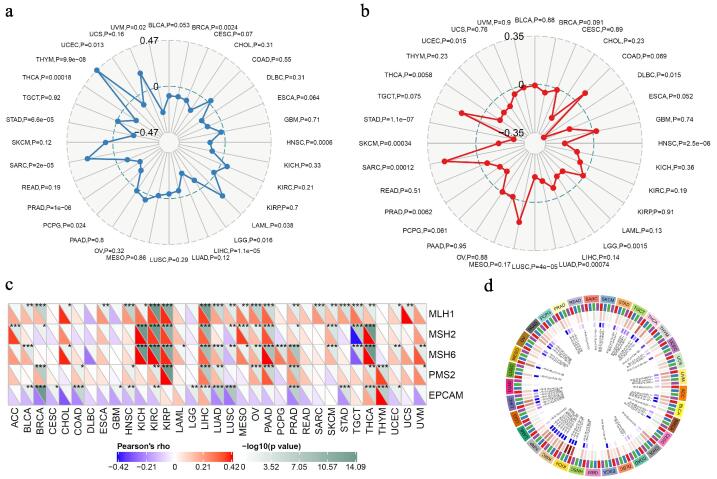
Correlation of AXL with Neoantigen, TMB and MSI. (a) The correlation of TMB with AXL expression in different types of cancers. (b) The correlation of MSI with AXL expression in different types of cancers. (c) The correlation of MMRs with AXL expression in different types of cancers. (d) The correlation of DNA methylated genes with AXL expression in different types of cancers.

To explore other potential mechanism of AXL in cancers. Neoantigens were specific antigens encoded by mutated genes of tumor cells, which could be used to design and synthesize neoantigen vaccines to achieve the effect of treating tumors [18]. Here we counted the number of neoantigens in each tumor sample separately, and found that AXL expression had significant correlation with and the neoantigen in BRCA, UCEC, PRAD, HNSC and STAD (Supplementary Fig. S4). In addition, the number of mutations in tumor cells were calculated by using TMB package in R. Among them, tumors with higher correlation coefficients were THYM, UCEC, UVM, BRCA, HNSC, LGG, LIHC, PCPG, PRAD, SARC, STAD and THCA (Fig. 6a). The MSI of UCEC, DLBC, HNSC, LGG, LUAD, LUSC, PRAD, SARC, SKCM, STAD and THCA had a certain relationship with AXL expression (Fig. 6b). Further, five DNA repair genes: MLH1, MSH2, MSH6, PMS2, EPCAM were used to assess the relationship between intracellular mismatch repair mutations and AXL expression and we found that KICH, KIRC, KIRP and PAAD had a high correlation with MMRs (Fig. 6c). The correlations of AXL with DNMT1, DNMT2, DNMT3A and DNMT3B were showed in Fig. 6d.

## 4 Discussion

The COVID-19 was still a major problem facing the world and it mainly caused respiratory diseases [19], [20]. The increasing number of SARS-CoV-2 virus infection cases suggested that this virus cloud affect almost all major organs in the human body, including the lungs, pharynx, heart, liver, brain, and kidneys. However, the ACE2 expression was all below 1% in other tissues [6]. Therefore, it was possible that there are more proteins other than ACE2 that influenced viral entry.

Some studies suggested that SARS-CoV-2 could utilize ACE2 or AXL to invade cells [8], [21]. Overexpression of AXL and ACE2 in human 293 T cells could effectively promote the entry of SARS-CoV-2 and knockout of AXL could significantly reduce the virus infection rate in H1299 lung cells and primary human lung epithelial cells [7]. As we known, AXL was widely expressed in almost all human organs and its expression was also much higher than that of ACE2. Our results further verified that AXL was highly expressed in the important organs affected by SARS-CoV-2, and its expression in the lung was significantly higher than that of ACE2. In addition, the expression of AXL in immune cells was also consistent with many current reports. Further, the difference in the expression of AXL protein in peripheral blood plasma of different gender individuals might also be one of the factors that cause the difference in susceptibility of different genders.

Studies have already proposed AXL as a therapeutic target against SARS-CoV-2, as well as its role in cancer [22], [23]. It has been reported that AXL was highly expressed in lung cancer and significantly increased the infection rate of SARS-Cov-2 in patients [20]. In this study, we found that AXL and its ligand GAS6 were highly expressed in many malignant tumors not only in lung cancer. AXL could bind to GAS6 and activate multiple pathways and participate in multiple processes of tumorigenesis, including regulating tumor cell growth, proliferation, migration, invasion and enhancement angiogenesis, etc [24]. Studies also showed that AXL was associated with resistance in patients to antitumor chemotherapy drugs (such as paclitaxel and cisplatin) or molecularly targeted drugs (such as erlotinib and gefitinib) [25], [26], [27]. AXL was also widely concerned as a drug target and the inhibitors against AXL mainly include small molecule inhibitors and monoclonal antibody drugs [28]. Other studies also showed that the AXL inhibitors could significantly inhibit tumor growth and improve survival in animal models of different tumors including chronic myeloid leukemia, non-small cell lung cancer and acute myeloid leukemia [10], [28], [29]. Here, our data suggest that AXL expression was strongly correlated with TMB, MSI, and the expression of DNA repair and methyltransferase genes in most tumor types. This might be one of the reasons why AXL protein could be used as a prognostic molecular marker for some tumors. AXL inhibitors could promote tumor immunity by modulating the polarization of macrophages. Our data indicated that the NK-cells, plasmacytoid dendritic cell, myeloid dendritic cell were highly expressed AXL and these cells were always considered to have an important relationship with the occurrence and development of tumors and the prognosis of tumor patients. In addition, our immune infiltration analysis also demonstrated that there was an inextricable link between AXL expression and immune scores in cancer patients, especially in BLCA, BRCA and CESC.

The current related antibody drugs designed based on AXL protein could become a new breakthrough in the clinical intervention of SARS-Cov-2 infection. The abnormal immune function of tumor patients and the existence of AXL-related tumor neoantigens suggested that there might be potential risks in the application of AXL-related tumor vaccines or SARS-Cov-2 vaccines in patients. However, the AXL protein as a new receptor brought new opportunities for the development of SARS-Cov-2 antibody drugs, but the uncertain mutation of the SARS-Cov-2 was bound to be another challenge in the development of antibody drugs.

## 5 Authors' contributions

WNZ, XPL, PFW, LZ and XHX analyzed and interpreted the data and wrote the manuscript. YJD, WNZ and XHX revised the manuscript.

## Declaration of Competing Interest

The authors declare that they have no known competing financial interests or personal relationships that could have appeared to influence the work reported in this paper.
