# Supplementary figures and images for: Comprehensive analysis of the novel omicron receptor AXL in cancers
Source: Comput Struct Biotechnol J. 2022 Jun 27;20:3304–12. doi: 10.1016/j.csbj.2022.06.051 (PMC9234055; doi:10.1016/j.csbj.2022.06.051)

a

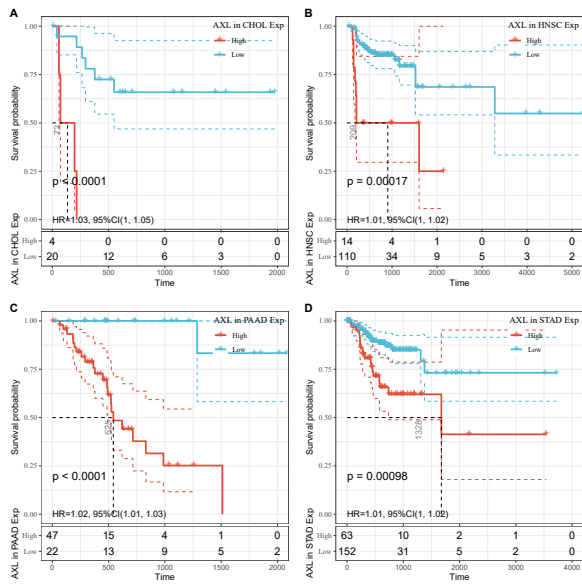

b

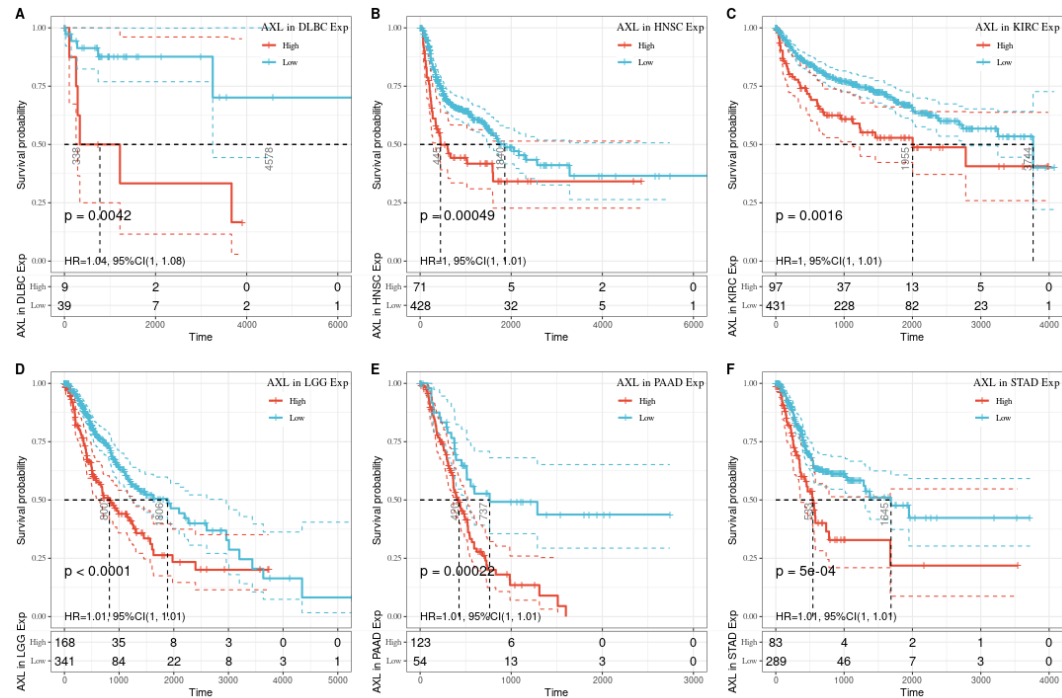

Supplement: Supplementary data 1 [file mmc1.pdf]

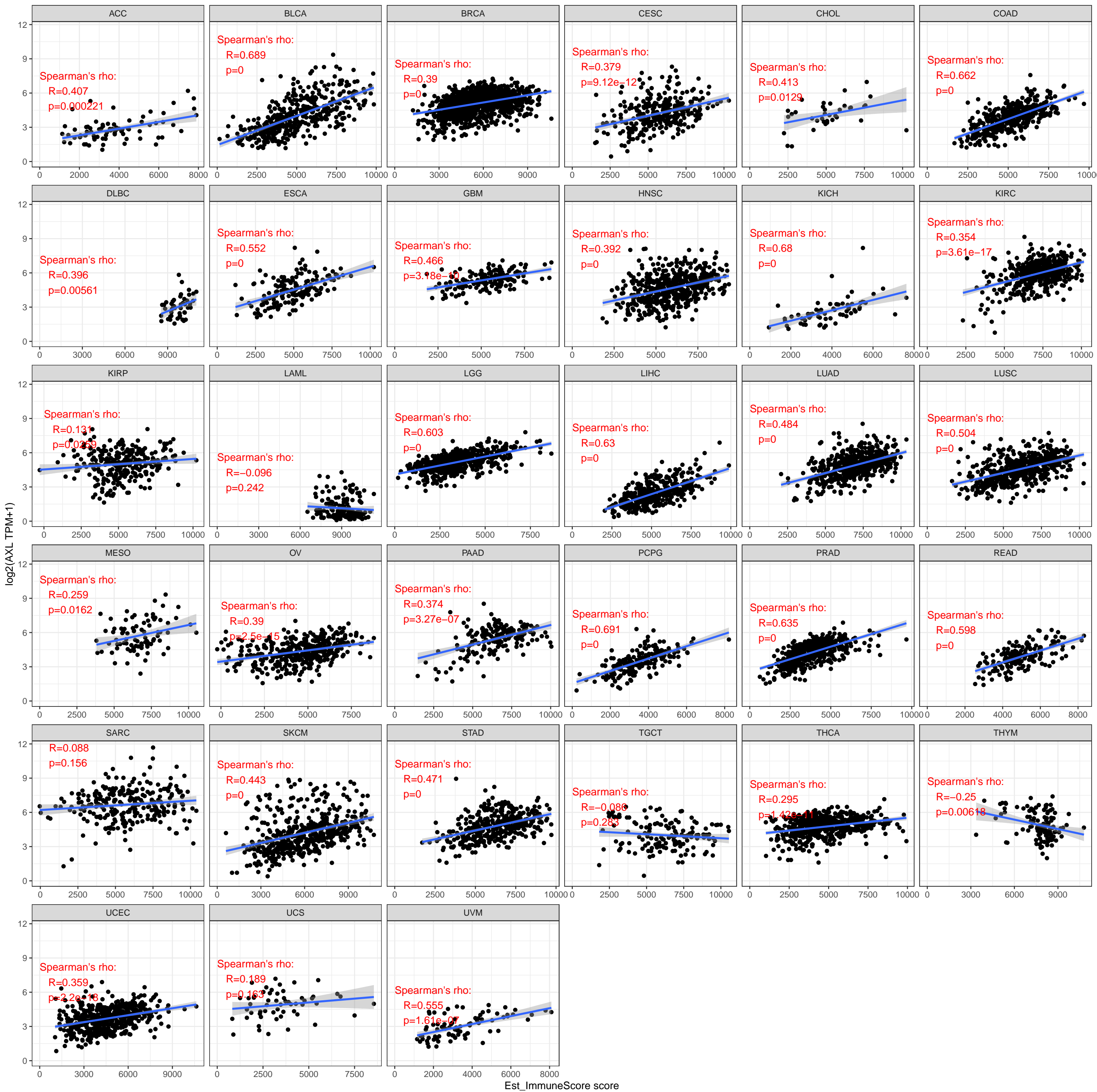

Supplement: Supplementary data 2 [file mmc2.pdf]

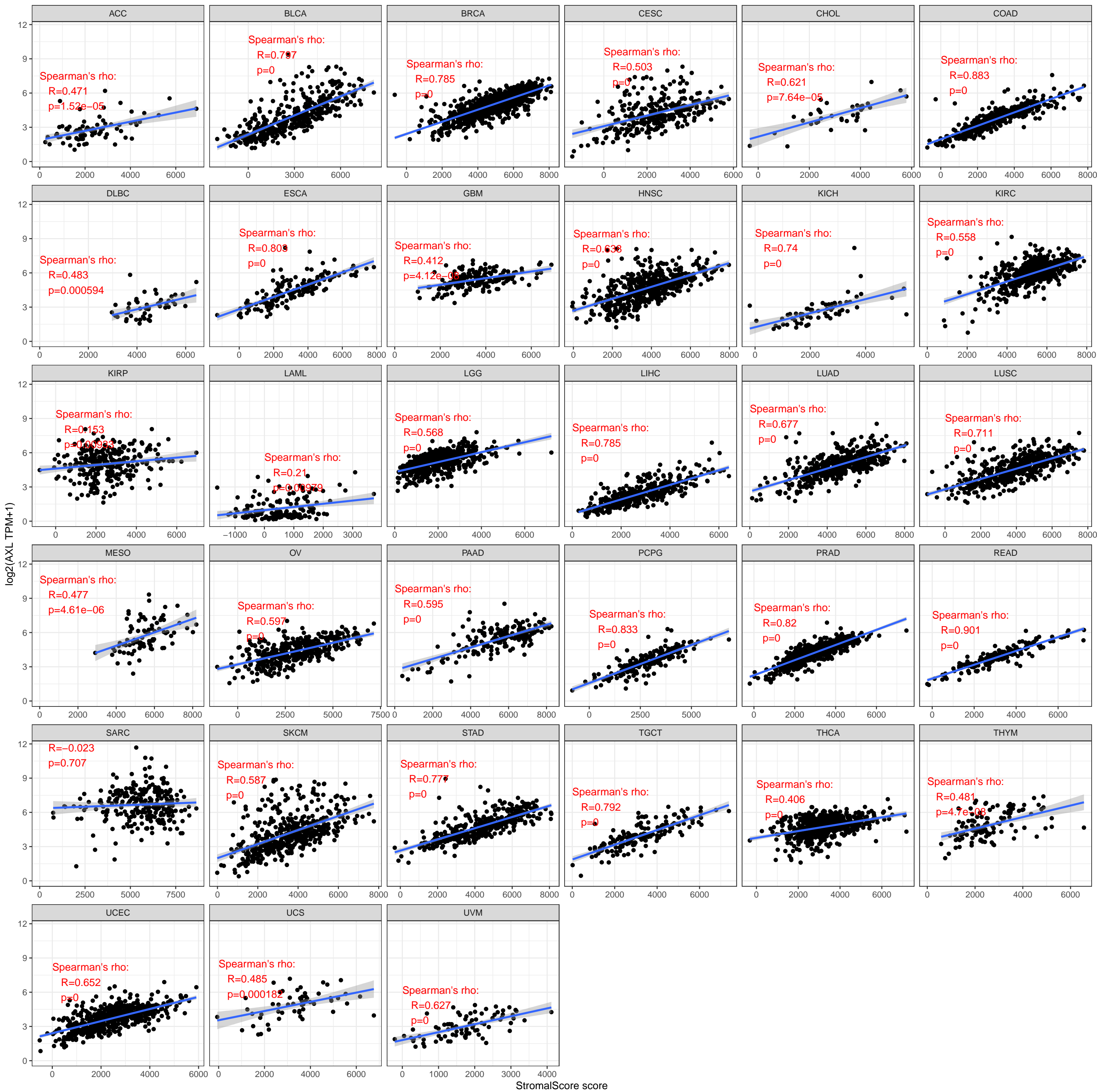

Supplement: Supplementary data 3 [file mmc3.pdf]

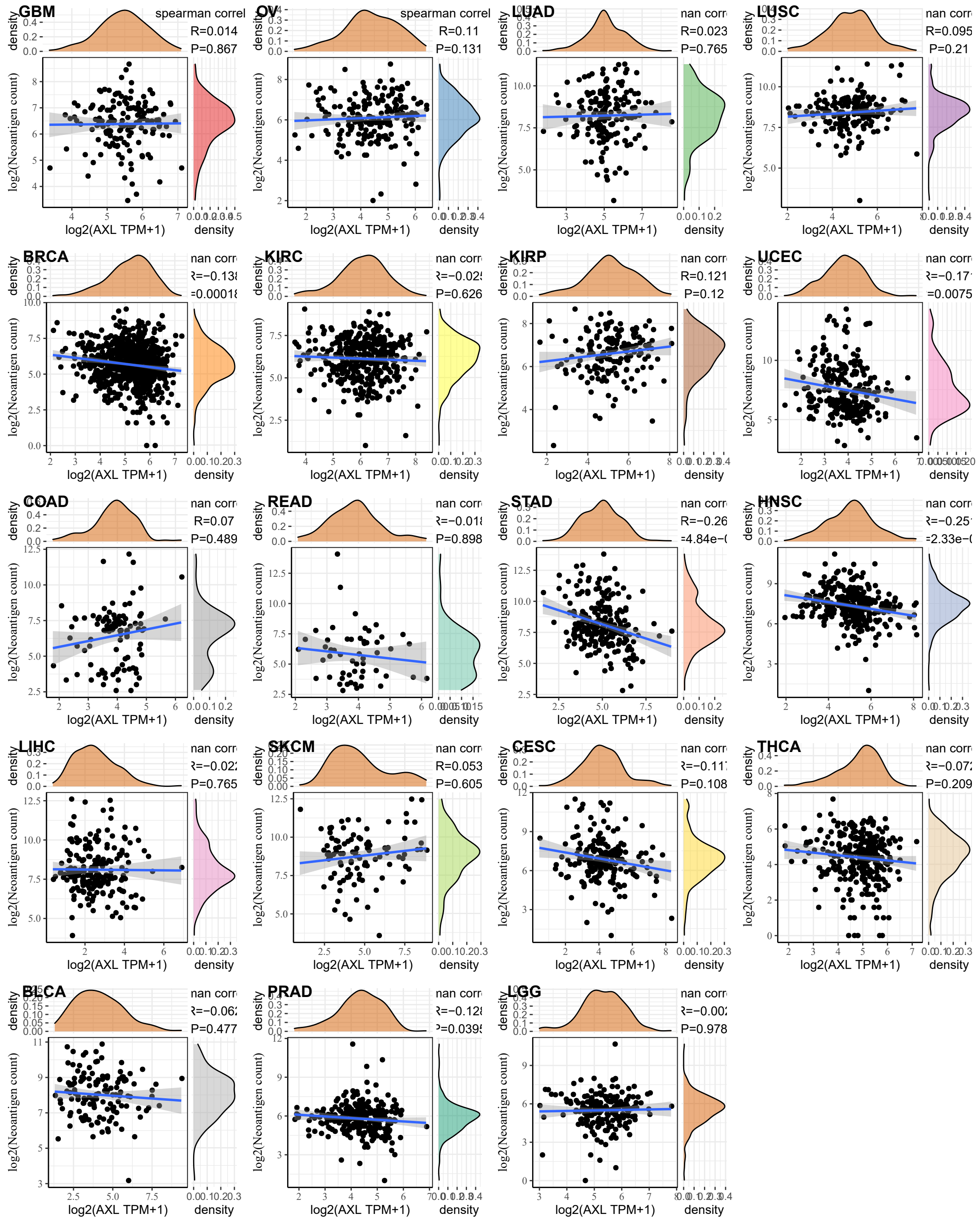

Supplement: Supplementary data 4 [file mmc4.pdf]
